# Secretome of *in vitro* cultured human embryos contains extracellular vesicles that are uptaken by the maternal side

**DOI:** 10.1038/s41598-017-05549-w

**Published:** 2017-07-12

**Authors:** Elisa Giacomini, Riccardo Vago, Ana Maria Sanchez, Paola Podini, Natasa Zarovni, Valentina Murdica, Roberta Rizzo, Daria Bortolotti, Massimo Candiani, Paola Viganò

**Affiliations:** 1 Reproductive Sciences Laboratory, Division of Genetics and Cell Biology, IRCCS San Raffaele Scientific Institute, Milano, 20132 Italy; 2 Urological Research Institute, IRCCS Ospedale San Raffaele, Milan, 20132 Italy; 3 Università Vita-Salute San Raffaele, Milan, 20132 Italy; 4 Department of Neuroscience, Institute of Experimental Neurology, IRCCS San Raffaele Scientific Institute, 20132 Milan, Italy; 5 Exosomics Siena S.p.A, Siena, 53100 Italy; 6 Department of Medical Sciences, Section of Microbiology and Medical Genetics, University of Ferrara, 44121 Ferrara, Italy; 7 Obstetrics and Gynecology Unit, IRCCS San Raffaele Scientific Institute, Milano, 20132 Italy

## Abstract

Communication between embryo and maternal endometrium occurs during a specific time frame in which implantation is possible. Here we demonstrate for the first time that conditioned media from non-manipulated human embryos cultured *in vitro* for 3 days or up to the blastocyst stage contain extracellular vesicles (EVs) with a diameter of 50 to 200 nm and bearing the traditional microvesicle and exosome marker proteins CD63, CD9 and ALIX. The embryonic origin of these EVs has been confirmed by the presence of stemness gene transcripts and their enrichment in the non-classical HLA-G protein. *NANOG* and *POU5F1* transcripts were shown to be contained in vesicles deriving from embryos at different stages of development. In line with a higher detection rate of the HLA-G protein in blastocysts compared to cleavage stage embryos, a significantly higher amount of HLA-G was found in vesicles accumulated in spent media from day 3 to day 5 of development compared to those isolated from the earlier stage. Uptake of dye-labeled embryo-derived EVs by human primary endometrial epithelial and stromal cells was also demonstrated with a fluorescence intensity signal significantly higher for cells treated with vesicles derived from blastocysts. Based on these findings, EV exchange may be suggested as an emerging way of communication at the maternal-fetal interface.

## Introduction

Since the first gestation reported in 1976^1^, more than five million pregnancies have been achieved worldwide by *in vitro* fertilization and its modifications, known generically as assisted reproductive technologies (ARTs). Currently, ART accounts for 1 to 3 percent of live births in the United States and Europe. Despite significant advances in the understanding of infertility mechanisms and the overcoming of many deficiencies in human fertility by evolving ART, the number of ‘take-home’ babies still remains low^2^. Research in this area is moving toward the improvement of success rates through a better understanding of embryo and uterine physiology^3^.

Embryo implantation and consequent pregnancy is thought to involve a two-way communication between maternal uterus and the blastocyst, a dialogue whose success seems essential for the progression through the processes of embryo apposition, adhesion, attachment and penetration^4–6^. Some embryonic signals modulating this dialogue have been identified^7–9^. Among them, human chorionic gonadotrophin synthesized early by the trophoblast cells acts on the uterine environment via the luteinizing hormone/hCG receptor and exerts both autocrine effects, promoting differentiation^10^ and migration of trophoblasts^11^, and paracrine effects on the maternal endometrium^12^. Another molecule identified in embryo culture media and supposed to be involved in the regulation of local maternal immune response is represented by sHLA-G^13^. HLA-G1/G5 protein expression has been detected in human preimplantation embryos in association with β2-microglobulin and the soluble spliced isoform has been proposed as a noninvasive tool for embryo selection in ART^14^. Very recently, miRNAs secreted by the embryos were suggested to be involved in endometrial cell growth and proliferation, proposing the existence of a previously unrecognized alternative communication system^15^. Acquisition of endometrial receptivity preceding blastocyst attachment is reflected by several cellular and ultrastructural changes, including gradual loss of uterine epithelial cell polarity, formation of apical surface pinopodes and the induction, even relatively unaffected by ovarian hormones, of a great number of locally expressed growth factors, cytokines, transcription factors, and vasoactive molecules. However, given the ethical restrictions limiting mechanistic studies, identification of embryonic signals promoting implantation remains so far elusive^5^.

Recently, increasing importance for all aspects of inter-cell communications is acknowledged to extracellular vesicles (EVs), heterogeneous populations of endogenous nano- and micro-sized cell-derived membrane vesicles released by eukaryotic and prokaryotic cells^16^. Their membranous shell prevents degradation of their contents, which comprise primarily soluble factors, proteins and RNAs, making possible long-duration and long-distance actions^17^. During cell binding and uptake, EVs induce a sort of functional expansion in the cell, transferring their functional transcriptome, proteome and lipidome to recipient cells and also inducing epigenetic modifications^18, 19^. Overall, there are plenty of evidence indicating that EV-shuttled biomolecules can profoundly affect the phenotype and activity of their target cells^20^.

EV secretion has been demonstrated for most cell types including embryonic stem cells and *in vitro* produced embryos derived from some mammalian species^21, 22^. However, to date no comprehensive data have been reported regarding human embryo-derived EVs. In this context, embryos grown *in vitro* during ART cycles offer a unique possibility to determine the presence of EVs in easily collectable embryonic secretome. We thus comprehensively characterized EVs secreted by human preimplantation embryos at different developmental stages and investigated their potential internalization by the maternal compartment. The results from this study provide hints for the EV exchange as an emerging way of communication at the maternal-fetal interface.

## Results

### Human embryos cultured *in vitro* release EVs

**Figure 1:**
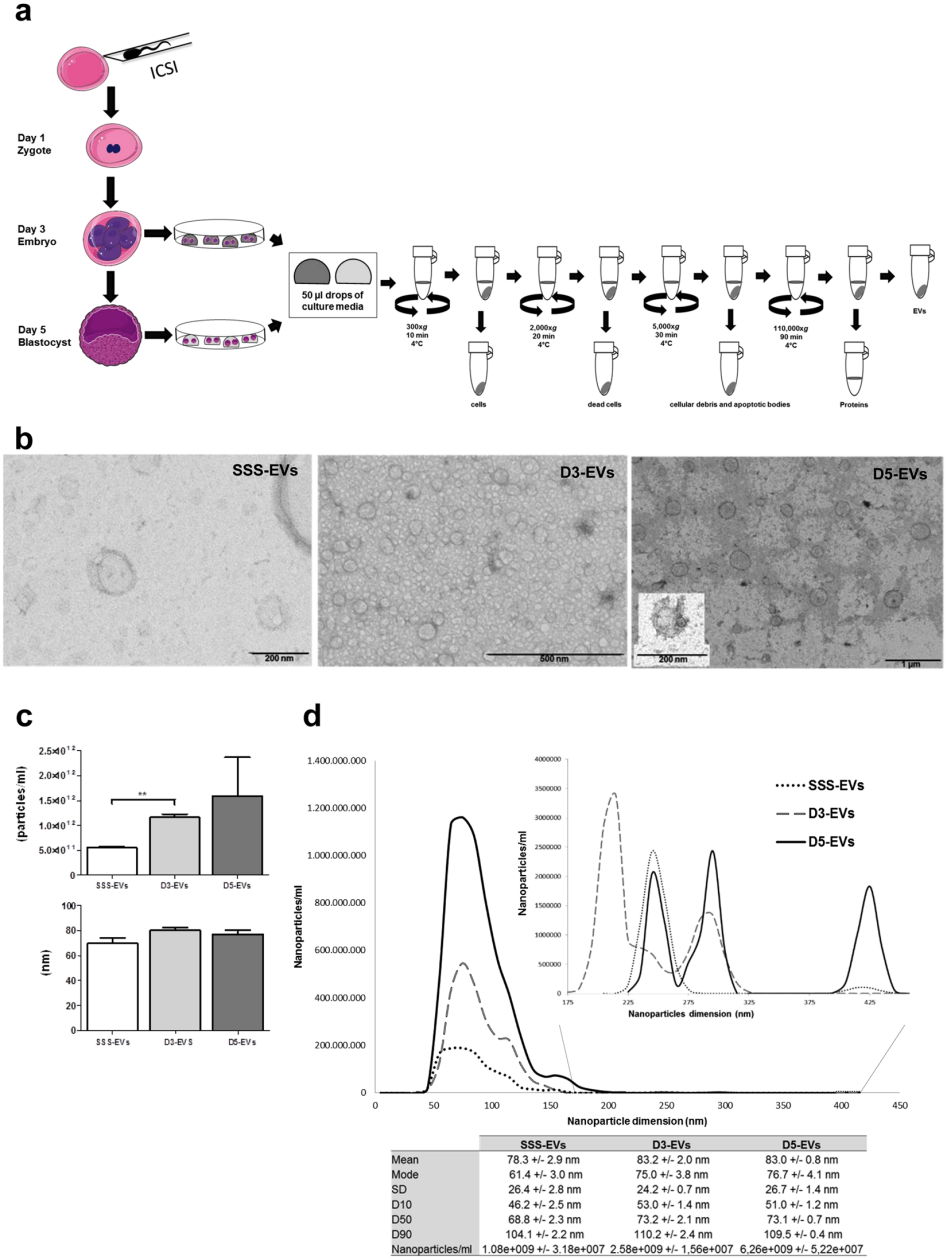
Day 3 and day 5 embryos release a heterogeneous population of EVs. (**a**) Schematic overview of the isolation protocol used to obtain EVs from embryo spent media accumulated from day 1 to day 3 (D3-EVs) or from day 3 to day 5 (D5-EVs) of development. (**b**) Fresh D3-EVs or D5-EVs isolated from six embryos were negatively stained with uranyl acetate and visualized by TEM. EVs isolated from SSS-supplemented medium (SSS-EVs) are also shown (left panel). Data shown are representative of 3 independent experiments. (**c**) Concentration defined as particle/ml (upper panel) and size distribution (lower panel) profiles were determined by NTA using 50 µl of spent media from six day 3 or day 5 embryos. Size and number of SSS-EVs are also shown. Results are expressed as mean ± SEM, n = 4 (**p = 0.001, D3-EVs vs SSS-EVs). (**d**) A representative tracking analysis plot of SSS-EVs (dilution 1:500) and of D3-EVs and D5-EVs (dilution 1:500). To better visualize peaks, a different scale was used for nanoparticles of diameter higher than 175 nm. The mode, mean value, standard deviation of size and concentration for each sample are provided. The value D50 represents the median size. Similarly, 90 percent of the distribution lies below the D90 value, and 10 percent of the population lies below the D10 value.

In ART practice, depending on how many embryos are produced and their quality, they can be kept in the laboratory for different days and are more frequently transferred back in the patient’s uterus after 3 or 5 days from fertilization. For isolation and analysis of EVs, the spent medium was collected from embryo cultures at day 3 (D3-EVs) or day 5 (D5-EVs) after fertilization. In both time points, media have been exposed to embryos for 48 hours. Conditioned medium from embryo group cultures of maximum 6 embryos was collected, pre-cleared of debris and subjected to a series of ultracentrifugation steps (Fig. 1a). Culture media alone, either additioned or not with 5% Human Serum Albumin (HSA) or with 10% Serum Substitute Supplement (SSS) were used as negative controls. Transmission electron microscopy (TEM) analysis of resuspended pellets from embryo-derived conditioned media revealed the presence of an heterogeneous population of membranous vesicles differing in size, shape and electron-density. Both D3-EVs and D5-EVs were in a size range from 50 to 200 nm with most of them smaller than 100 nm (Fig. 1b). This finding was in line with results from nanoparticle tracking analysis (NTA) of 50 µl of embryo-derived conditioned medium showing similar size distribution profiles for the D3-EV and D5-EV populations, with a diameter of 80.38 ± 2.16 nm and 77 ± 3.16 nm (mean ± SEM), respectively (Fig. 1c). The evaluation of the ninety percent of nanoparticles distribution (D90) allowed to confirm that the absolute majority of vesicles in D3-EVs and D5-EVs population laid in the <110 nm range. The small number of particles detected by NTA in culture media alone (similar to that found in PBS) or in HSA-supplemented media was not shown to be vesicles in nature as confirmed by TEM analysis that did not reveal any presence of EVs in culture media alone and hardly any in HSA-supplemented media (HSA-EVs, <1/TEM field) (data not shown). Conversely, medium supplemented with SSS as a FDA-approved protein source for embryo culture, contained EVs (SSS-EVs) with a diameter ranging from 50 to 200 nm, an average size of 69.4 ± 4.48 nm and a D90 below 94.3 ± 5.00 nm (Fig. 1b,c). NTA measurement did also demonstrate a statistically significant increased number of EVs in spent media from day 3 embryos compared to those present in SSS-supplemented medium (p < 0.001). A higher number of vesicles was also found in spent media from day 5 embryos compared to SSS-supplemented medium but with a much greater variability among the independent experiments (Fig. 1c,d).

### EV marker analysis of human embryo-derived vesicles confirms a standard protein composition for EVs

**Figure 2:**
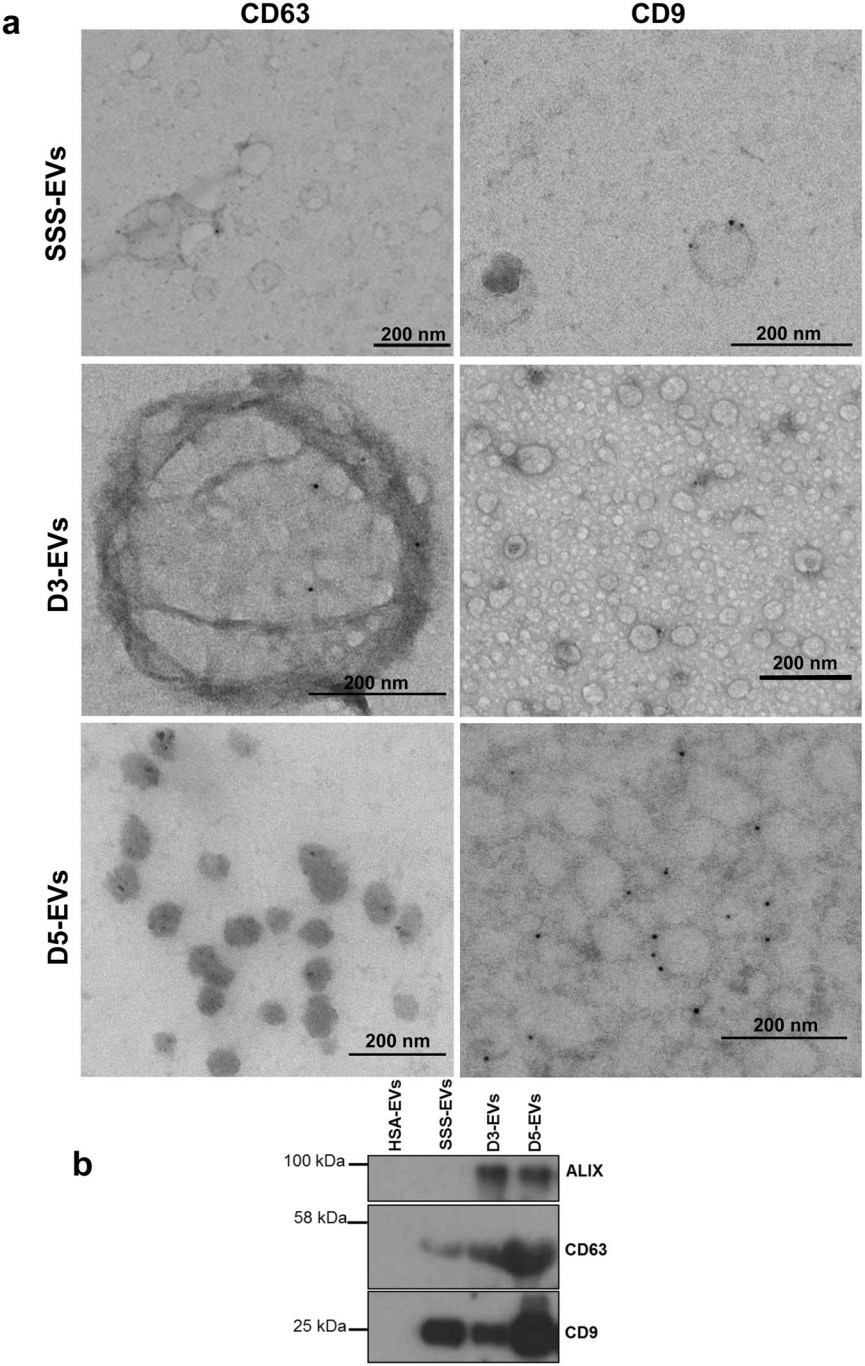
Marker characterization of EVs released by day 3 and day 5 embryos. (**a**) EVs isolated from a single drop of spent media from six embryos at day 3 (D3-EVs) or day 5 (D5-EVs) developmental stage were negatively stained with uranyl acetate and visualized by TEM after immunogold labelling with anti-CD63 and anti-CD9 antibodies. Transmission electron microscopy images of EVs from SSS-supplemented medium (SSS-EVs) after immunogold labelling with anti-CD9 and anti-CD63 is also shown (upper panels). Black dots indicate membrane-associated gold. Data shown are representative of 3 independent experiments. (**b**) Ten µg of proteins isolated from D3-EVs and D5-EVs and from an equal volume of fresh 10% SSS- or 5% HSA-supplemented media were analyzed for the presence of molecular markers of microvesicles and of exosomes by western blotting. Data shown are representative of 3 independent experiments.

To further confirm that the structures observed in the ultracentrifugation pellet were indeed EVs according to the guidelines of the International Society for Extracellular Vesicles^23^, characterization was performed by electron microscopy immunogold staining and western blotting analysis. Immunoelectron microscopy allowed to confirm the presence of EVs with CD9 and CD63 immunoreactivity in spent media from embryo at different developmental stages (Fig. 2a) and also in SSS-supplemented media (Fig. 2a upper panel), while EVs were not detectable in culture media alone and in HSA-supplemented media (data not shown). Western blotting demonstrated the presence of CD9 and CD63 markers in D3-EVs, D5-EVs as well as in SSS-EVs but not in HSA-EVs. The exosome marker protein ALIX was detected in both D3-EVs and D5-EVs but not in SSS-EVs confirming the presence of bona fide exosomes in the EV preparations from embryo secretome (Fig. 2b).

### Human embryo-derived vesicles contain mRNAs for embryonic pluripotency-related genes and HLA-G protein

**Figure 3:**
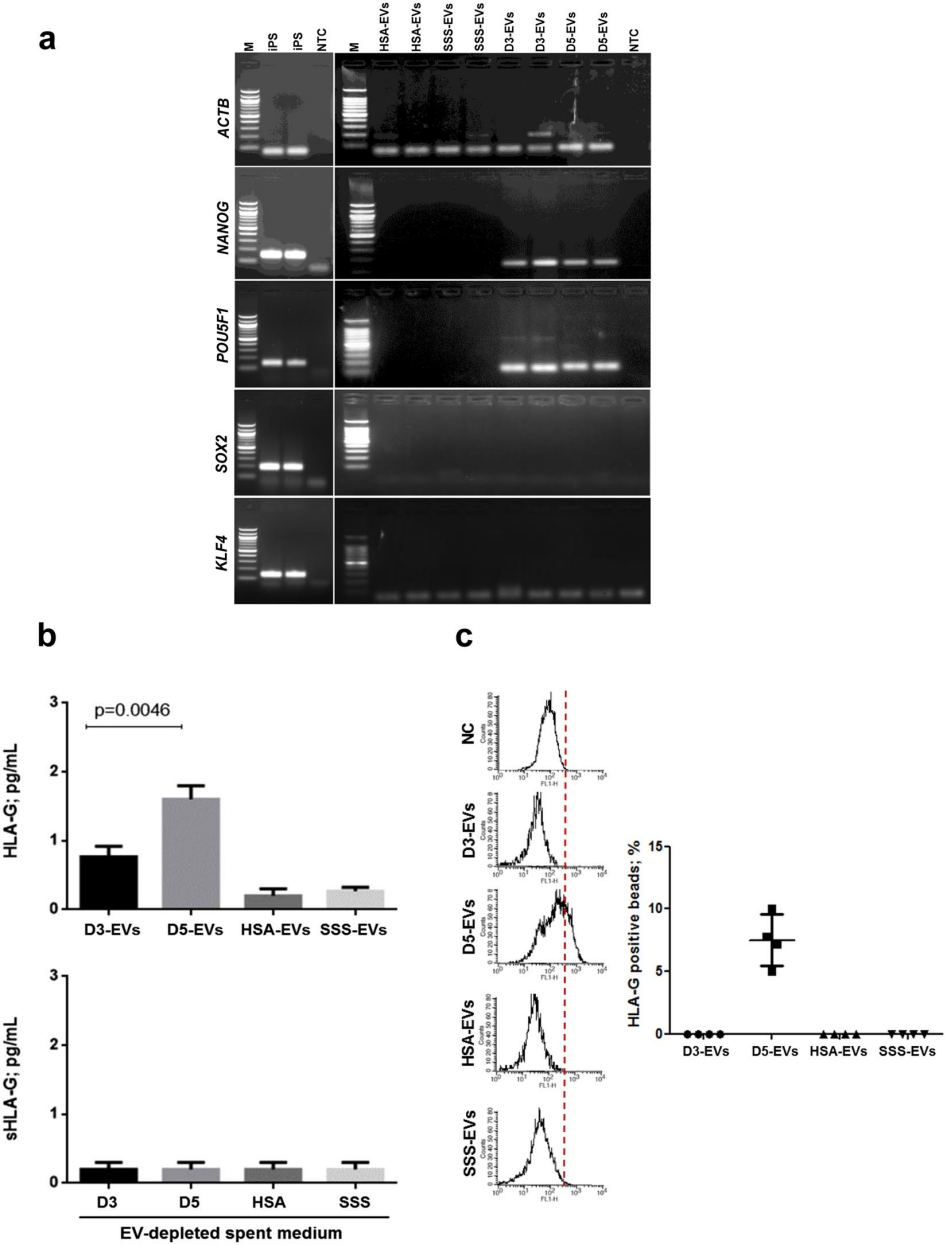
EVs isolated from day 3 and day 5 embryos contain embryonic specific molecules. (**a**) Five ng of EVs-derived amplified cDNA from a pool of spent media from 50 day 3 (D3-EVs) or day 5 (D5-EVs) embryos were subjected to touch down PCR in order to assess presence of pluripotency-related gene transcripts. Gel electrophoresis of PCR products is shown. iPS cDNA was used as a positive control and EVs derived from fresh media conditioned with 10% of SSS or 5% of HSA were used as negative controls (HSA-EVs and SSS-EVs). No template controls (NTC) were also added to distinguish the primer dimer formation. Three independent experiments have been performed and two different samples per condition are shown. M = 100 bp molecular ladder. (**b**) HLA-G levels in EVs (upper panel) from a pool of spent media of 50 embryos in day 3 (D3-EVs) or day 5 (D5-EVs) of development were evaluated by Bio-Plex assay. Equal volumes of fresh media conditioned with 10% of SSS (SSS-EVs or SSS) or 5% of HSA (HSA-EVs or HSA) were used as negative controls. Results are expressed as mean ± SEM, n = 6 (p < 0.0046, D5-EVs vs D3-EVs). Soluble HLA-G levels have been also evaluated in EV-depleted spent culture media from the same samples by Bio-Plex assay, n = 3 (lower panel). (**c**) Flow cytometry analysis was performed to confirm the levels of HLA-G in EVs. The same analysis was performed in both EVs and in transfected HeLa-G5 cell culture supernatants used as positive control (not shown) and in HeLa wild-type cell culture supernatants as negative control (NC). The samples were analyzed in duplicates. Results are expressed as mean ± SEM, n = 4.

mRNA contents for pluripotency-related genes (*POU5F1*, *SOX2*, *KLF4* and *NANOG*) were evaluated in D3-EVs and D5-EVs (Fig. 3a) by using neuronal iPS cells as a positive control and HSA-EVs and SSS-EVs as negative controls. The PCR normalized on the EVs-derived amplified cDNA quantity (5 ng per each sample) showed that *ACTB* transcript was present in all the samples evaluated, including an evident amount in HSA-EVs and SSS-EVs. *POU5F1* and *NANOG* mRNA band could be detected in both D3-EVs and D5-EVs suggesting the presence of both transcripts in EVs from embryos at different developmental stages (Fig. 3a). *SOX2* and *KLF4* transcripts were not detected (Fig. 3a). EVs isolated from both HSA- and SSS-supplemented media resulted negative for the all pluripotency-related gene transcripts evaluated.

Results of HLA-G detection in D3-EVs, D5-EVs and in HSA-EVs and SSS-EVs as negative controls are shown in Fig. 3b. Bio-plex and cytometry analyses were performed using MEM-G/9 moAb-conjugated beads which are specific for HLA-G1, shed sHLA-G1 and sHLA-G5 (soluble counterpart of HLA-G1). Detectable levels of HLA-G were not found in HSA-EVs and SSS-EVs while they were found in D5-EVs samples (1.6 ± 0.08 pg/ml; mean ± SEM) in a statistically significantly higher amount compared to D3-EVs samples (0.7 ± 0.11 pg/ml; mean ± SEM) (p < 0.0046) (Fig. 3b upper panel). This observation was confirmed by flow cytometry analysis demonstrating a mean percentage of 8.1 ± 2.3 of total beads positive for HLA-G signal in D5-EVs samples while D3-EVs and EVs from negative controls resulted completely negative (Fig. 3c). The detection limits for flow cytometry have been fixed using negative controls (HeLa wild-type cell culture supernatants) and positive controls (transfected HeLa-G5 cell culture supernatants). In EV-depleted spent culture media deriving from each sample (Fig. 3b lower panel), sHLA-G levels were always under the Bio-Plex detection limit.

### Human embryo-derived EVs are taken up by primary endometrial cells (ECs)

**Figure 4:**
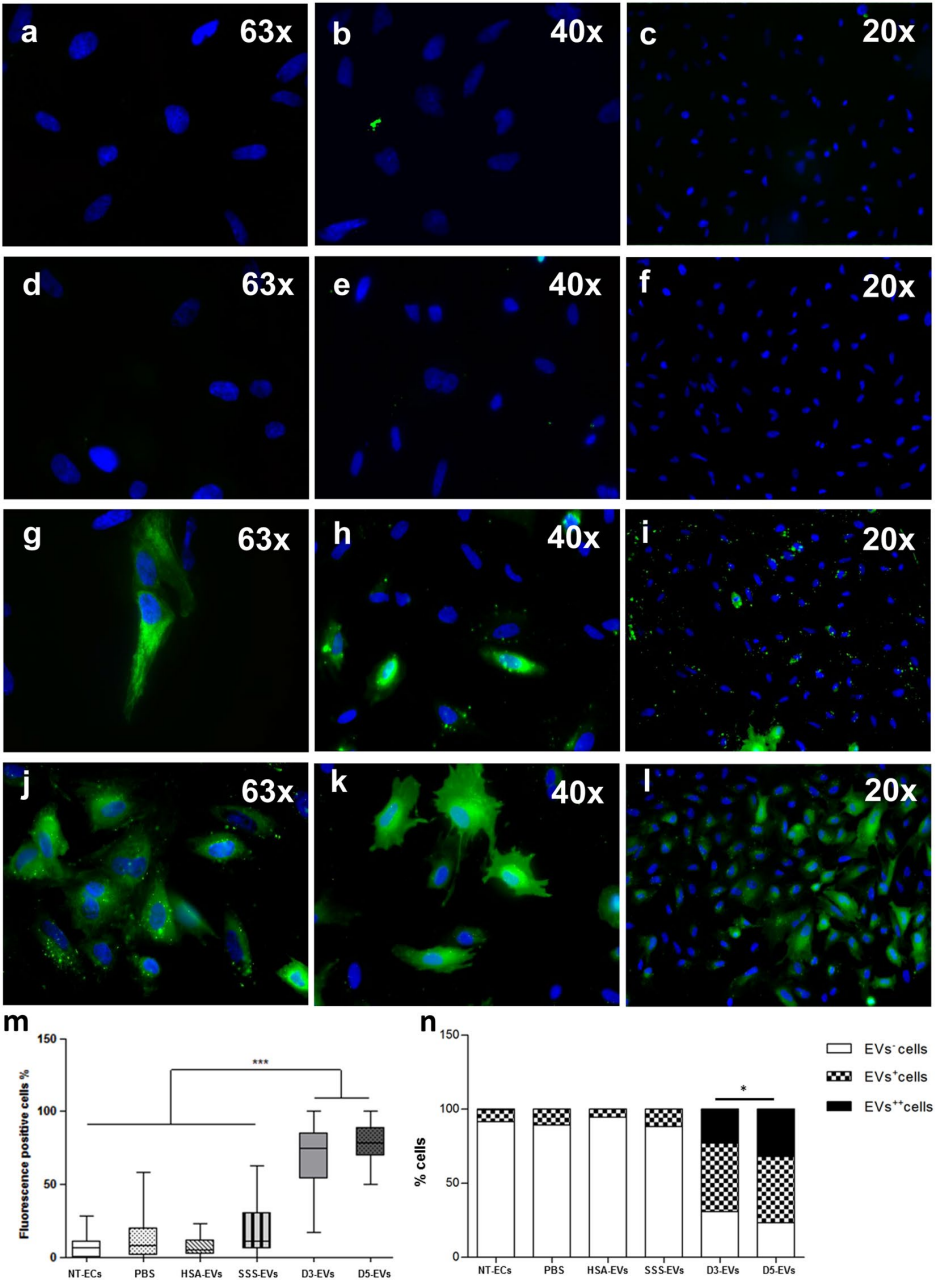
Primary ECs can incorporate embryo-derived EVs. Representative images of primary ECs treated for 4 hours with 10 µg/ml of Vybrant DiO-labelled EVs derived from 5% HSA-supplemented fresh medium (HSA-EVs) (**a**–**c**), from 10% SSS-supplemented fresh media (SSS-EVs) (**d**–**f**) and from conditioned media of day 3 embryos (D3-EVs) (**g**–**i**) and of day 5 embryos (D5-EVs) (**j**–**l**). Blue = nuclei. Box plot of percentage of dye-positive cells (EV^+^ plus EV^++^cells). Block lines within boxes represent median value and the whiskers indicate the minimum and the maximum [N = 3, ***P < 0.0001, D3-EVs/D5-EVs vs all negative controls (PBS, HSA-EVs and SSS-EVs)] (**m**); engrafted distributions of fluorescence intensity among the different EV species used for primary cell treatment (*P = 0.01, D3-EVs vs D5-EVs) (**n**).

In order to examine whether EVs derived from embryos could be uptaken by primary ECs and thus potentially establish a communication with the maternal side, D3-EVs and D5-EVs were labeled with Vybrant DiO carbocyanine dye (green fluorescence) as described in the Methods section. Vybrant DiO-labeled D3-EVs and D5-EVs were incubated with ECs for 4 hours followed by cell visualization using fluorescence microscopy (Fig. 4g–l). Cells co-incubated with the three negative controls (PBS, as well as HSA-EVs and SSS-EVs labeled in the same manner) showed no signals or very low signals (Fig. 4a–f). After treatment with D3-EVs and D5-EVs, percentages of green fluorescence positive cells (EV^+^ plus EV^++^ cells, 68.81 ± 4.85 and 78.31 ± 2.89, respectively; mean ± SEM) were statistically higher compared to those relative to the negative controls (p < 0.0001; D3-EVs vs all negative controls; p < 0.0001; D5-EVs vs all negative controls) (Fig. 4m). Although the percentage of positive cells was similar between samples uptaking D3-EVs or D5-EVs, based on the scoring system used, the intensity (Fig. 4n) and the subcellular distribution pattern (Fig. 4g–l) of the signal were significantly different between the two conditions (p = 0.01). In particular, cells treated with D3-EVs exhibited a spotted fluorescent pattern while those treated with D5-EVs demonstrated a more diffused fluorescence suggesting a faster uptake mechanism for EVs derived from spent media of day 5 embryos. Uptake of D3-EVs and D5-EVs has been also demonstrated when the separated components of the endometrium, the epithelial and the stromal cells, have been used for the analysis (Supplementary Fig. 1a). Finally, a faster uptake mechanism for D5-EVs was also confirmed based on a time-course evaluation of endometrial stromal cells treated with D3-EVs and D5-EVs (Supplementary Fig. 1b)

## Discussion

The preimplantation mammalian embryo is currently known to be relatively autonomous as a remarkable self-organizing capacity has been demonstrated in the blastocyst. Indeed, the key hallmarks of human embryogenesis, including the cellular polarization leading to the formation of pro-amniotic cavity, take place in the absence of any maternal tissues^24^. Nevertheless, embryos can influence their microenvironments through secretion of autocrine/paracrine factors constituting the ‘embryo secretome’. Relevance of this environment is demonstrated by the increased success of *in vitro* culture when embryos are kept in groups and this is valid for different mammalian species^25, 26^. Moreover, over the years evidence has emerged supporting the idea that factors of embryonic origin may favour attachment of the blastocyst to the uterine lining and invasion into the uterus^27^. The nature of the key embryo secretome factors implicated in these phenomena is however still to be completely elucidated.

For the first time, we have herein demonstrated that human preimplantation embryos at different developmental stages can release EVs during their *in vitro* culture for ART procedures. Our findings show EVs to be CD63^+^, CD9^+^ and ALIX^+^ suggesting their predominant exosomal nature although the presence of other EVs, such as microvesicles, is likely. Indeed, a limited population of EVs with a diameter higher than 100 nm could be observed in analyzed samples. The embryonic origin of these EVs has been confirmed by the presence of stemness gene transcripts and their enrichment in HLA-G protein.

The expression of early lineage-specific genes in pre-implantation embryos can vary significantly between species, with implications for developmental control and stem cell derivation. In humans, the pluripotency-associated transcription factor *POU5F1* is initially expressed in 8-cell embryos at 3 days post-fertilization. *POU5F1* expression remains high in both the inner cell mass (ICM) and trophectoderm (TE) in Day 5 early-blastocysts and is primarily restricted to the ICM by Day 6. *NANOG* is expressed in human preimplantation embryos and its expression is restricted to the ICM earlier than *POU5F1* so that there is no evidence of *NANOG* localization in the TE at day 5 stage or later in day 6 embryos^28^. Based on our results, both *POU5F1* and *NANOG* mRNA could be detected in EVs from both day 3 and day 5 embryos consistent with the idea that in spent media from blastocysts in culture, EVs may derive not only from TE as the external and more consistent layer but also from the ICM. Conversely, EVs isolated from embryos cultured *in vitro* did not show positivity for *SOX2* transcripts whose expression in human embryos is localized largely to ICM nuclei of expanded human blastocysts, in all blastomeres in early blastocysts and in early stages. KLF4 that does not localize to a specific compartment within the primate embryo was similarly not expressed in isolated EVs^29, 30^. Thus, it seems that only some members of the transcription network regulating stemness are privileged in EVs derived from human embryos.

The detection of HLA-G molecules in EVs isolated from embryo spent culture media represents further evidence for their embryonic origin. Indeed, the expression of HLA-G in blastocysts and embryos in various cleavage stages has been reported in different studies^31–35^. HLA-G has been found to be strongly present in the TE of blastocysts, in TE hatching from the zona pellucida, and in TE projections^31^ supporting the idea that HLA-G could be implicated in initial attachment and implantation of the embryo in the maternal uterus. HLA-G has been also detected in the ICM with a prevalence in the outer cells (future hypoblast)^32^. Two observations may derive from our results in this regard: (i) in embryo spent culture media following removal of EVs, the protein, as revealed using the MEM-G/9 antibody which is specific for HLA-G1, shed sHLA-G1 and sHLA-G5, was under the detection limit. This finding would suggest that, for the most part, HLA-G that has been reported to be present in embryo secretome by various authors^31–35^ was indeed associated with EVs. This would suggest that it is not a simple shed protein but supports further its potential role as a mediator of the dialogue with the maternal side; (ii) the protein was mainly detected in EVs deriving from spent culture medium accumulated from day 3 to day 5 (D5-EVs). The low or undetectable amount of HLA-G in EVs derived from culture medium accumulated from day 1 to day 3 (D3-EVs) could be explained by the HLA-G origin. Indeed, HLA-G detected in day 3 embryo culture medium may derive from maternal source of EVs whereas HLA-G detected in culture media of day 5 blastocysts could be neosynthetized and released as associated with EV membranes. Since in humans, activation of embryonic genome occurs between days 2 and day 3 of development after second cleavage division^36^, the possibility that maternal protein expression of HLA-G may be carried over from the oocyte and be implicated in early HLA-G activity in the embryo has been previously considered^35^. This hypothesis for a different origin of HLA-G in culture medium from embryos at different developmental stages is supported by Yao *et al*.^34^ that did not observe mRNA expression for *sHLA*-*G5* in cleavage stage embryos and detected it only in 20% of the blastocysts analyzed while *HLA*-*G1* mRNA was observed in only 20% of cleavage stage embryos and in 80% of blastocysts. In any case, available evidence support a higher detection rate of HLA-G protein in blastocysts compared to cleavage stage embryos^31, 34^, in line with our findings in corresponding EVs.

The observation of EV release by the preimplantation embryo in other (non-human) species is not novel. Interestingly, a recent report has demonstrated that mouse embryonic stem cells can shed EVs that use the extracellular matrix proteins fibronectin and laminin alpha5 to bind their receptors on trophoblasts. This would engage FAK and JNK signaling pathways thereby promoting trophoblast migration^21^. Moreover, when these vesicles were injected into blastocysts, they markedly enhanced the ability of blastocysts to undergo implantation. Porcine parthenogenetic embryos were shown to secrete EVs that can be internalized by nuclear transfer embryos^22^. Although informative on the ability of embryonic cells to generate EVs, these studies have used cellular models generated *in vitro*. The fact that non-manipulated human preimplantation embryos, even in the early stages, can indeed release exosomes represents an important step for the understanding of the full breadth of roles played by EVs in reproductive and stem cell biology.

Importantly, EVs were also present in the protein supplement routinely used in the embryo culture media. Serum Substitute Supplement, in particular, is derived from the pooled heat-inactivated sera of human donors. After heat inactivation, fractions of human albumin and α-, β-, and γ-globulins are purified and freeze-dried. The powdered components are dissolved in physiological saline to yield concentrations of 84% albumin, 16% α- and β-globulins and <1% γ -globulins. Based on EV size, it is possible that they readily pass through the 200 nm filtration process the manufacturer uses for sterilization. This is somehow alarming considering that recent studies have suggested that EVs from fetal bovine serum (FBS) could substantially influence cultured cell behavior and phenotype since vesicle-depleted FBS have reduced capacity to support cell growth. FBS-derived vesicles have been shown to mediate anchorage-independent growth of breast carcinoma cells and cultured muscle cells grown in EV-depleted FBS were unable to differentiate and stopped proliferating after few passages^37^. Although embryos are not cultured in FBS, some of the protein supplements used in ART procedures are native substances. They are prepared throughout FDA-approved procedures from a purified fraction of serum that should contain no lipid component or other non-protein plasma constituents^38^. By adding commercial SSS to the embryo culture medium there is an additional 0.5% (w/v) increase in final albumin concentration and a 0.1% addition of globulins (w/v). SSS added to commercial HSA-supplemented human embryo culture media was shown to result in an overall increase in implantation and live birth rates when the embryo transfer occurred on day 5^39^. Reasons for these improved results are presently unclear as not all constituents of serum supplements are known. Moreover, the nature and concentration of these unidentified constituents seem to be product- and batch-specific. For these reasons, the demonstration of the presence of EVs in these serum supplements opens a new era of investigation in relation to the safety of IVF culture media as recently underlined by Sunde and coworkers^40^.

On the other hand, in our experimental conditions, EVs present in the protein supplements could not be up-taken by human endometrial cells. Indeed, following confirmation of EV presence in the secretome of human embryos and their biomolecular characterization, we have evaluated the incorporation of these vesicles by the natural counterpart, the endometrium. EVs labeled with Vybrant DiO fluorescent dye were co-cultured with monolayers of primary human endometrial cells and an effective uptake of embryo-derived EVs was demonstrated thus suggesting that only these vesicles can be internalized by endometrial cells using a specific mechanism of recognition. In mice, embryonic cells release EVs which can activate signalling pathways in trophoblasts, leading to enhanced migration into the uterus^21^. We will explore whether a similar mechanism of communication is also engaged with maternal cells. The higher intensity of the incorporated fluorescent signal when endometrial cells were treated with EVs derived from day 5 blastocysts compared to those from day 3 embryos would hint for a role of vesicles in the modification of implantation events occurring at the blastocyst stage such as adhesion and migration^41^. Importantly in this regard, a two-way communication via EVs seems to be in place at the maternal-embryo interface. In humans, exosomes derived from receptive phase endometrium have been shown to be internalized by trophoblasts thus enhancing their adhesive capacity partly through active focal adhesion kinase signalling^42^. Finally, we have demonstrated that both the components of the endometrium, the epithelial and the stromal cells, were able to uptake embryo-derived EVs. Thus, involvement of EVs in other aspects of endometrial physiology such as decidualization cannot be excluded and is worthy of further investigation.

Finally, the findings presented herein may have outbreaking consequences from a clinical point of view. Indeed, since EVs contain specific information from the embryo that also recapitulate its developmental process in time and are transmitted to the maternal side, the simple process of media changing in the various steps from fertilization to embryo transfer could be viewed with a completely different strategy approach. The idea of embryo grouped cultures or to use a single medium throughout the culture instead of sequential media according to embryo developmental stages should be revisited in this regard.

In conclusion, one of the main cause of failure during ART procedures is the inability of the embryo to implant. Our results showing that during *in vitro* culture for ART human embryos can secrete EVs that can be easily uptaken by the maternal side raise some exciting possibilities regarding their potential therapeutic use as a co-factor for promoting the establishment of a successful pregnancy.

## Methods

### ART Procedures and Human Embryo Spent Medium Collection

The embryo spent culture medium samples employed in this study were obtained from patients attending the Centro Scienze Natalità of the San Raffaele Hospital in Milano, Italy, with an indication to ART. All embryos were obtained after controlled ovarian stimulation that was performed using a gonadotropin-releasing hormone-agonist or antagonist protocol. Oocyte collection was performed 36 hours after triggering of ovulation. After 2–3 hours incubation in 5% HSA-supplemented Fertilization medium (Sage *In*-*Vitro* Fertilization, Inc. Trumbull, CT, USA) under oil, selected oocytes were allocated to conventional *in vitro* fertilization or ICSI. For ICSI, denudation of the cumulus oophorus was performed as previously described^26, 43–45^. Fertilized oocytes were sequentially cultured in group cultures of maximum 6 embryos in 50-µL microdrops of Quinn’s Advantage Cleavage medium (Sage In-VitroFertilization Inc., Cooper Surgical, Trumbull, CT, USA) supplemented with 10% SSS (Irvine, CA, USA) in a humidified atmosphere containing 5% O_2_ and 6% CO_2_. On day 3 after fertilization, cleavage-stage embryos were transferred to fresh 50-µL drop of Quinn’s Advantage Blastocyst Medium (Sage In-VitroFertilization Inc., Cooper Surgical, Trumbull, CT, USA) supplemented with 10% SSS. The quality of the cleavage stage embryos and of the day 5 blastocysts was assessed and defined according to standard criteria^26, 44, 45^. The spent culture medium was collected from embryos at the cleavage stage or from expanded blastocysts without signs of degenerating cells during ART cycles and kept in −80 °C until use. The study (GFI2015) was approved by the Ethics Commitee of the Ospedale San Raffaele in Milan, Istituto di Ricovero e Cura a Carattere Scientifico. Informed consent was obtained from all participants, and the experiments were performed in accordance to the principles set out in the World Medical Association Declaration of Helsinki.

### Isolation of EVs from spent media of human embryos at different development stages

Spent culture medium samples from embryos on day 3 or day 5 of development were subjected to differential centrifugation according to previous protocols^46, 47^. The same protocol of EVs isolation was used for all samples considered in this study. Briefly, in order to remove cells, dead cells, and cellular debris, conditioned media of embryos/blastocysts were centrifuged at 300 x *g* for 10 min, at 2,000 x *g* for 20 min and at 5,000 x *g* for 30 min; all centrifuges were performed at 4 °C. The supernatants were transferred in a 3,5-mL thick wall polycarbonate tubes (Beckman Coulter Inc., Brea, CA, USA, item no. 349622) and an equal amount of PBS was added. The sample was ultra-centrifuged at 110,000× *g* for 90 min at 4 °C in a TLA-100.3 rotor (Beckman Coulter Inc.) using Optima TLX centrifuge (Beckman Coulter Inc.) to pellet EVs. The supernatants were removed completely, thereby pellets were washed with 1 mL of PBS and centrifuged 90 min at 110,000 x *g* at 4 °C. The pellets containing embryo-derived EVs accumulated from day 1 to day 3 (D3-EVs) or from day 3 to day 5 (D5-EVs) of development were suspended in an adequate volume of PBS (50–100 µl) and the protein concentration was measured by Bradford assay and by NanoDrop8000 to assess EVs purification. We ensured that only samples with a protein content of at least 10 µg were utilized for the downstream experiments. The final EVs pellet was stored at −80 °C until use. Fresh media supplemented with 10% SSS or 5% HSA were subjected to the same procedures to ensure proper negative controls to each experiment.

### Nanoparticle tracking analysis

Nanoparticle tracking analysis (NTA) was performed by an expert EV laboratory (Exosomics Siena S.p.A, Siena) in order to provide a validation of the results. NTA was performed using a NanoSight LM10-HS microscope (NanoSight Ltd., Amesbury, UK), as previously described^48^. For the analysis, 50 µl of spent media deriving from 6 Day 3 embryos, 50 µl of spent media of 6 Day 5 blastocysts, 50 µl of fresh media completed with 10% SSS and 50 µl of fresh media with 5% of HSA were diluted with concentrations adjusted, if necessary, in order to specifically fit the optimal working range (20–50 particles per frame) of the instrument. Three 60-s videos were recorded under the flow mode for each sample with camera level set at 16 and detection threshold set at 10. At least 200 completed tracks per video were collected. The camera level and overall settings were selected as optimal for the accurate detection of exosome-sized nanoparticles following the manufacturer recommendations and as confirmed during instrument calibration. Videos were analyzed with NTA software version 2.3 to determine the mean, mode and estimated concentration of measured particles with corresponding standard error. The NanoSight system was calibrated with polystyrene latex microbeads of 50, 100, and 200 nm (Thermo Scientific Waltham, MA, USA) prior to analysis and auto settings were used for blur, minimum track length and minimum expected particle size.

### Transmission electron microscopy

Preparation for transmission electron microscopy (TEM) analysis was done using the method described by Théry *et al*.^46^_._ EVs derived from single drops of spent media from six embryos at day 3 stage (D3-EVs), day 5 blastocyst stage (D5-EVs) and from 10% SSS or 5% HSA fresh media were mixed with an equal volume of 4% PFA and deposited by airfuge onto Formvar/Carbon coated EM grids (Ted Pella, Redding, CA, USA). Samples were contrasted and embedded by treatment with uranyl-oxalate solution (Electron Microscopy Science, Hatfield, PA, USA), pH 7, for 5 min, followed by methyl-cellulose-uranyl-acetate (Sigma, Saint Louis, MO, USA) on ice for 10 min. Negative stain immunogold labeling was carried out exclusively on fresh EVs. The live specimens in suspension were mounted onto formvar, carbon-coated grids, and allowed to adhere to the grids. The specimens were then exposed to the primary antibody followed by 10 nm gold-labelled secondary antibody (British Biocell, Cardiff, UK). The following primary antibodies were used: anti-CD9 (10 µg/ml; BD Pharmingen, #555370, San Jose, CA, USA) and anti-CD63 (10 µg/ml; BD Pharmingen, #556019, San Jose, CA, USA). After washing to eliminate any non-specific binding of either the primary or secondary antibody, the specimens were fixed using 2.5% glutaraldehyde and contrasted in 2% uranyl acetate and lead citrate. LEO 912AB Omega electron microscope (Carl Zeiss NTS, Oberkochen, Germany) was used for image analysis.

### Western blot analysis

D3-EVs and D5-EVs were isolated from a pool of spent media from 50 embryos at different developmental stage and from an equal volume of 10% SSS- or 5% HSA- supplemented fresh media as negative controls and were used for immunoblotting analysis. Ten µg of isolated EVs (by Bradford assay and by NanoDrop8000 measurement) were lysed in reducing sample buffer [0.25 M Tris–HCl (pH 6.8), 40% glycerol, 8% SDS, 5% 2-mercaptoethanol and 0.04% bromophenol blue] and boiled for 5 minutes at 95 °C. For tetraspanins detection, non-reducing sample buffer (without 2-mercaptoethanol) was used. Proteins were resolved by SDS-PAGE (SDS-polyacrylamide gel electrophoresis), electrophoretically transferred to polyvinylidene fluoride membranes, blocked in 5% non-fat powdered milk in TBS-T (0.5% Tween-20) and the membranes were incubated with the following antibodies: anti-ALIX (1:500, Santa Cruz, #sc-271975, Santa Cruz, CA, USA), anti-CD9 (1:1000, BD Pharmingen, #555370, San Jose, CA, USA), and anti-CD63 (1:1000; BD Pharmingen, #556019, San Jose, CA, USA). Protein bands were detected using X-ray film and enhanced chemiluminescence reagent (ECL, Amersham, Buckinghamshire, UK).

### RNA extraction, Reverse transcription and Whole Transcriptome Amplification

Since the RNA content was expected to be limited, D3-EVs and D5-EVs isolated from a pool of spent media from 50 embryos at different developmental stage and an equal volume of 10% SSS- or 5% HSA- supplemented media as negative controls, were subjected to RNA extraction, reverse transcription and whole transcriptome amplification using REPLI-g Cell whole genome amplification (WGA) & whole transcriptome amplification (WTA) Kit (Qiagen, Hilden, Germany) according to the manufacturer’s instructions. Briefly, lysis buffer was added to 13 µl of EVs resuspended in PBS and heated at 95 °C for 5 min to lyse and release the EV contents. Lysed EVs were used for WTA of total RNA. Genomic DNA was removed, cDNA was synthesized and subjected to ligation and amplification steps. The RT-reaction was included in the kit which used T-Script reverse transcriptase combined with random and oligo-dT primers. REPLI-g SensiPhi DNA Polymerase with high proofreading was used for isothermal amplification of cDNA. The DNA derived from amplification of cDNA was stored at −80 °C or immediately used. To avoid contamination among samples, we adopted precautions normally used during routine viral diagnostic PCR analysis at SSI, where extraction, amplification and analyses were physically separated and negative samples were included in all steps.

### Touch-down PCR of pluripotency-related genes

EV-derived amplified cDNA from spent medium of embryos at different development stages was used for the detection of pluripotency gene transcripts. In order to quantify and perform the PCR amplification using the same amount of cDNAs from each sample, purification of amplified DNA by LiCl/EtOH precipitation was performed, according to supplementary protocol instructions of REPLI-g kit. In order to ensure optimal normalization of results on total amplified cDNA quantity, 5 ng of the amplified cDNA of EVs isolated from embryo spent medium were subjected to PCR using AmpliTaq Gold® DNA Polymerase, LD (low DNA) (Applied Biosystems, Foster City, CA, USA). The PCR amplification was carried out for one cycle of denaturation at 95 °C for 10 min and subsequent 40 cycles. To increase specificity, sensitivity and yield of amplification, a touch-down PCR was performed starting from an annealing temperature of 72 °C to 57 °C over the course of 15 cycles, followed by 25 standard cycles with denaturation at 95 °C, annealing and extension at 60 °C for 30 sec. Primer sequences are listed in Supplementary Table S1. Ten microliters of PCR products were loaded on a 2% agarose gel and stained with EtBr. The positive control was cDNA of human neuronal iPS kindly provided from Dott. Giacomo Frati and Dott. ssa Angela Gritti (San Raffaele Telethon Institute for Gene Therapy)^49^ and the negative controls were amplified cDNAs from EVs from fresh media supplemented with 10% of SSS or with 5% of HSA.

### HLA-G detection: Bio-Plex system

Covalent coupling of antibodies to microsphere and HLA-G Bio-Plex assay were performed as described previously^50^. Briefly, covalent coupling of the anti-HLAG antibodies, MEM-G9 moAb (Exbio, Prague, Czech Republic) to the carboxylated polystyrene microspheres (Bio-Rad, Hercules, CA, USA) has been performed using the Bio-Plex amine coupling kit (Bio-Rad). Five μl of HLA-G standards assayed in duplicate or sonicated D5-EVs and D3-EVs from a pool of spent media from 50 embryos at different developmental stage have been incubated with 50 μl of anti-HLA-G conjugated beads (5000 beads/well) in 96-well filter plates for 60 min at room temperature with shaking. An equal volume of 10% SSS or 5% HSA supplemented media as negative controls was used. For the analysis of sHLA-G not associated with EVs, the same procedure was applied to 50 µl of 110,000 x *g* supernatant (EV-depleted spent culture media) of each sample. Plates have been washed by vacuum filtration with 100 μl of Bio-Plex wash buffer, 25 μl of biotinylated antibody W6/32 (10 µg/ml) (Dako, Glostrup, Denmark) has been added, and plates were incubated for 30 min at room temperature with shaking. After filter washes, 50 µl of streptavidin-phycoerythrin has been added, and the plates have been incubated for 15 min at room temperature with shaking. Finally, plates have been washed by vacuum filtration, beads have been suspended in 125 µl of Bio-Plex assay buffer, and samples have been analyzed on the Instrument Bio-Plex system in combination with the Bio-Plex Manager software. The standard curves for HLA-G have been used from 100 to 0.5 pg/ml and the minimum detectable dose was 1.0 pg/ml. The specificity of this assay has been validated with an isotype control (Mouse IgG1 Isotype control, code 1B-457-C100 biotin, Exbio, Praha, Czech Republic) used in place of W6/32 biotin moAb. The background observed was lower than the selected detection limit (data not shown). The intra-assay coefficient of variation (CV) was 1.4% and the inter-assay CV was 2.0%. The limit of detection is 0.5 pg/ml.

### HLA-G detection: Flow cytometry assay

The flow cytometry technique was also used to analyze the presence of HLA-G protein^51^ in EVs. Dynabeads M-280 Tosylactivated (Dynal Biotech, Oslo, Norway) were prepared according to the manufacturer’s recommendations. Briefly, the dynabeads were incubated for 24 hours at 37 °C with MEM-G9 MoAb (Exbio, Prague, Czech Republic), with a ratio of 10^7^ dynabeads to 3 μg MEM-G9 MoAb. After several washes in recommended buffers, the Dynabead-MEM-G9 conjugates were stored in PBS, pH7.4 with 0.1% BSA and 0.02% sodium azide at 4 °C. Transfected HeLa-G5 cell culture supernatants were used as positive control, and HeLa wild-type cells culture supernatants as negative control. After sonication, the EV content was incubated with 10^6^ dynabeads-MEM-G9 conjugated for 1 hour at 37 °C, then washed twice with PBS and labeled with anti-beta2microglobulin-FITC MoAb (Biodesign International, Saco, MN, USA) for 15 min at room temperature. The flow cytometric assay was performed on FACS CantoII (Becton Dickinson, San Jose, CA, USA) using standard settings and data analysis performed with Cell Quest software (Becton Dickinson).

### Establishing primary endometrial cell cultures from human endometrial samples

Human endometrial samples were obtained from women undergoing laparoscopy for ovarian benign pathology using a surgical curette. Women with previous autoimmune or neoplastic disorders were excluded from the study. Moreover, they were not taking oral contraceptives, progestins, GnRH analogues/antagonists or other hormonal medications. All subjects were <40 years of age and had regular menstrual cycles. All samples of uterine endometrium were obtained at the time of laparoscopy after informed consent was given. Endometrial tissues were used to establish primary endometrial cultures^52^. Cytofluorimetric analysis showed that leukocyte contamination (CD45-positive cells) of our cultures was less than 2%. Briefly, using a scalpel, the tissue was transected into small pieces (1–2 mm^3^) and washed in fresh medium to remove mucus or debris. Tissue was then incubated 2 hours at 37 °C in a shaking water bath in 0.2% of collagenase A (Hoffmann-La Roche, Basel, Switzerland) diluted in RPMI-1640 supplemented with 10% FBS and 1% penicillin/streptomycin (Lonza, Basel, Switzerland). The digested suspension was filtered through a 45 *μ*m pore sterile filter to remove undigested tissue. The endometrial cell suspension, containing mainly stromal and epithelial glandular endometrial cells, was centrifuged at 300 xg for 5 min and fresh media was added to the pelleted cells. Some experiments were also performed dividing the two components of endometrium, the epithelial and the stromal cells, according to a previous protocol^52^ including differential sedimentation and selective attachment to the dishes. The cells were finally plated for EV uptake assay. Endometrial cells were cultured for a maximum of 7–8 days.

### Labelling of EVs with Fluorescent Dye and EV Uptake Assay

EVs-D3 and EVs-D5 were labelled with the Vybrant™ DiO cell-labeling solution (#V22886, Molecular Probes, Eugene, OR, USA) following the procedures described by Nazarenko and colleagues^53^ with some modifications. Briefly, 100 μg of EVs sedimented by ultracentrifugation were resuspended in 200 μl of cold PBS. One µl of dye was diluted to 200 µl EV suspension and incubated 30 min at 37 °C by rotation. EVs labelled were purified by ultracentrifugation at 110,000 × *g* for 1 hour at 4 °C. Vybrant DiO-labelled EVs (5 µg, 10 µg/ml, EV proteins by Bradford assay and by NanoDrop8000 measurement) were added to 8 × 10^4^ ECs plated on glass coverslips onto 24-wells. Co-cultures were maintained at 37 °C for different time periods (30 min, 1, 2 and 4 hours). EV-containing medium was then removed and the cells were washed gently with 2X PBS to eliminate unbound EVs. The ECs were then fixed in 4% paraformaldehyde for 20 min at room temperature and washed three times with PBS. Then, coverslips with treated cells were mounted on a microscope slide with VECTASHIELD Antifade Mounting Medium with DAPI (Vector Laboratories, Burlingame, CA, USA) carefully avoiding building of air bubbles. As negative controls, PBS mixed to the dye, labelled HSA-EVs or SSS-EVs were processed similarly to other samples and added to culture wells. Non-treated endometrial cells (NT-ECs) were also used as a further negative control in order to exclude potential autofluorescence. Internalization of EVs was observed by immunofluorescence microscopy and virtual images were generated using the Axio Vision Imaging Software (Axiovision Rel 4.8^®^) on an Axio Imager M2 microscope (Carl Zeiss, Oberkochen, Germany). For standardization and evaluation of image quality, at least 10 images were randomly acquired by using a Zeiss color AxioCam MR5. All fluorescence images were captured using a fixed exposure time of 250 ms. The cells that internalized Vybrant DiO-labelled EVs were quantified analyzing the intensity of signal which was scored as EV^−^ cells (no signal), EV^+^ cells (moderate signal: fluorescent dots) and EV^++^ cells (strong signal: cytoplasmic diffusion of fluorescence).

## Statistical Analysis

All experiments were repeated with at least three independent biological replicates. Student’s *t* test, one-way ANOVA with Newman-Kuels multiple comparison tests and nonparametric Kruskal–Wallis test followed by the Dunn’s Multiple comparison’s tests were used as appropriate. Contingency table for fluorescence-intensity distributions was performed by chi-square test. All results are expressed as mean ± SEM or as median (range). All the analyses and relative graphs were made in Prism 5.0 (GraphPad Software Inc., La Jolla, CA, USA).

## Electronic supplementary material

Supplementary Information
