# Supplementary material for: Secretome of in vitro cultured human embryos contains extracellular vesicles that are uptaken by the maternal side
Source: Sci Rep. 2017 Jul 12;7:5210. doi: 10.1038/s41598-017-05549-w (PMC5507879; doi:10.1038/s41598-017-05549-w)
Supplement: Supplementary file 1 — Supplementary Information [file 41598_2017_5549_MOESM1_ESM.pdf]

Secretome of *in vitro* cultured human embryos contains extracellular vesicles that are uptaken by the maternal side

Elisa Giacomini [1], Riccardo Vago [2,3], Ana Maria Sanchez [1], Paola Podini [4], Natasa Zarovni [5], Valentina Murdica[2], Roberta Rizzo [6], Daria Bortolotti[6], Massimo Candiani [3,7] and Paola Viganò[1,*]

[1]Reproductive Sciences Laboratory, Division of Genetics and Cell Biology, IRCCS San Raffaele Scientific Institute, Milano,20132, Italy.

[2]Urological Research Institute, IRCCS Ospedale San Raffaele, Milan, 20132, Italy

[3]Università Vita-Salute San Raffaele, Milan, 20132, Italy

[4]Department of Neuroscience, Institute of Experimental Neurology, IRCCS San Raffaele Scientific Institute, 20132 Milan, Italy

[5]Exosomics Siena S.p.A, Siena, 53100, Italy

[6]Department of Medical Sciences, Section of Microbiology and Medical Genetics, University of Ferrara, 44121, Ferrara, Italy.

[7]Obstetrics and Gynecology Unit, IRCCS San Raffaele Scientific Institute, Milano, 20132, Italy.

Corresponding author*:

Viganò Paola, PhD

Reproductive Sciences Laboratory, Division of Genetics and Cell Biology, IRCCS San Raffaele Scientific Institute,

Via Olgettina, 60, 20132, Milano, Italy

Tel: +39 02 2346 6228

Fax: + 39 02 26434311

E-mail: vigano.paola@hsr.it

SUPPLEMENTARY INFORMATION

Supplementary Table S1. Sequences of primers used in this study.

Gene	Sequence (5'-3')	Product size (bp)
ACTB Forward	GGCACCCAGCACAATGAAG	66
ACTB Reverse	CCGATCCACACGGAGTACTTG	
NANOG Forward	ACCTTGGCTGCCGTCTCTGG	151
NANOG Reverse	TGTTTGGGATTGGGAGGCTTTGCT	
POU5F1 Forward	TTTTGGTACCCCAGGCTATG	136
POU5F1 Reverse	ATTCAAACTGAGGTGCCTGC	
SOX2 Forward	GAGCTTTGCAGGAAGTTTGC	190
SOX2 Reverse	GCAAGAAGCCTCTCCTTGAA	
KLF4 Forward	GCAGCCACCTGGCGAGTCTG	130
KLF4 Reverse	CCCCGAATAACCGCTGGCGG	

Supplementary Figure S1

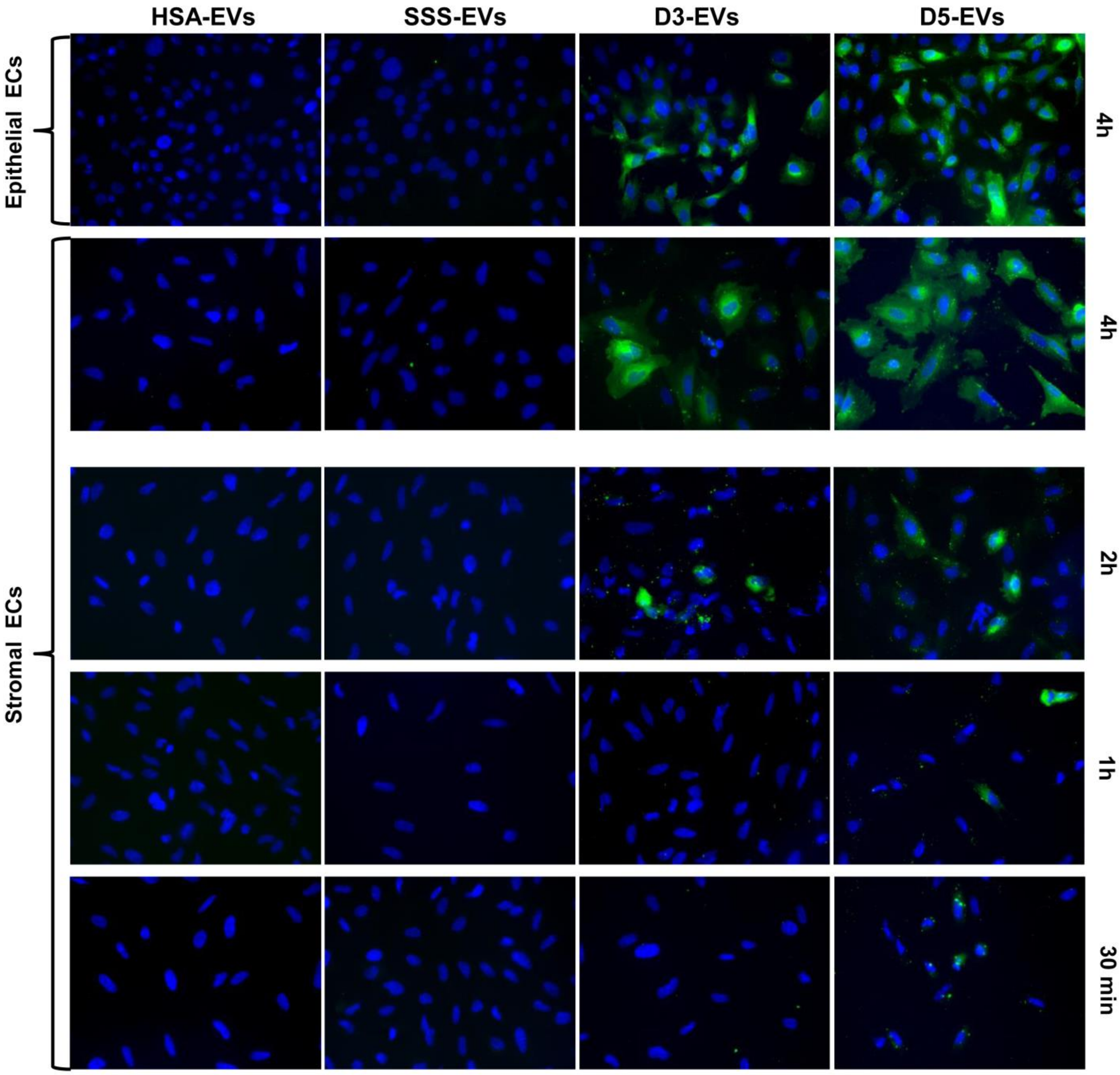

Supplementary Figure S1. Epithelial and stromal ECs can incorporate embryo-derived EVs. Representative images of primary epithelial and stromal ECs treated for 4 hours with 10 µg/ml of Vybrant DiO-labelled EVs derived from 5% HSA-supplemented fresh medium (HSA-EVs), from 10% SSS-supplemented fresh media (SSS-EVs) and from conditioned media of day 3 embryos (D3-EVs) and of day 5 embryos (D5-EVs). Endometrial stromal cells were also treated for different time (30 minutes, 1, 2 or 4 hours) with 10 µg/ml of Vybrant DiO-labelled EVs derived from 5% HSA-supplemented fresh medium (HSA-EVs), from 10% SSS-supplemented fresh media (SSS-EVs) and from conditioned media of day 3 embryos (D3-EVs) and of day 5 embryos (D5-EVs)with D3-EVs and D5-EVs. Blue = nuclei.
